# Biallelic OSM deficiency presents with juvenile myelodysplastic syndrome and response to treatment

**DOI:** 10.1172/JCI192422

**Published:** 2025-05-01

**Authors:** Abdullah H. Alfalah, Alfadil Haroon, Ahmed Alfares, Syed Osman Ahmed, Sateesh Maddirevula

**Affiliations:** 1 Precision Medicine Laboratory Department, Genomic Medicine Center of Excellence (GMCoE), King Faisal Specialist Hospital and Research Center, Riyadh, Saudi Arabia.; 2 College of Medicine, Jouf University, Pediatrics Department Aljouf, Sakaka, Saudi Arabi.; 3 Department of Hematology, Stem Cell Transplantation and Cellular Therapy, King Faisal Specialist Hospital and Research Center, Riyadh, Saudi Arabia.; 4 College of Medicine, Alfaisal University, Riyadh, Saudi Arabia.

**Keywords:** Genetics, Hematology, Bone marrow, Bone marrow transplantation, Genetic diseases

**To the Editor:** Garrigue et al. identified a biallelic variant in the human oncostatin M (OSM) gene in a consanguineous family (3 patients) with inherited severe bone marrow failure syndromes (IBMFS) in the patients aged from 10–20 years, with 4.4 years median age of onset, and in vitro and in vivo experiments established OSM roles in hematopoiesis (1). Similarly, we have identified three patients from 2 families with thrombocytopenia, anemia, and pancytopenia progressed to bone marrow failure with abnormal hematological values. Three affected females are homozygous for a loss-of-function variant (NM_020530.6: c.289C>T; Gln97Ter) in *OSM* (Figure 1A). They are aged between 17 years and 46 years, with the age of 14 being the disease onset median age, with a notable difference in the disease progression. The eldest patient (family 1; II:4) progressed to myelodysplastic syndrome (MDS), as she was suspected of having dyskeratosis congenita due to short telomeres requiring bone marrow transplant at the age of 40 years, with initial challenges due to transplant-related complications; however, the condition is stabilized with a successful outcome. This progression was not noted in family II (II:6 and II:9). It is worth noting the disorder may lead to MDS as the disease progresses.

Remarkably, two patients (family II; II:6 and II:9) in our study have responded to eltrombopag and danazol with an increase in hemoglobin, white blood cells, and platelets (Figure 1B) from the seventh week of the treatment (Table 1). Our observation of eltrombopag response provides direct evidence of treatment for OSM deficiency. However, eltrombopag is an alternative to hematopoietic stem-cell transplantation (HSCT) in OSM deficiency and it, in turn requires a longer follow-up, and its mechanism needs to be investigated. Here, we reported three females with a founder variant with early truncation (Gln97Ter) in *OSM*. Hypothetically, early truncation can cause a severe phenotype; however, we noticed a late onset of disease in our patient. This could be because of IL-6 family genes sharing functional overlap with OSM (2). The findings from additional families (*n* = 3) with founder *OSM* variant establish the genotype-phenotype of *OSM* in bone marrow failure syndrome, elucidate the disease progression with a different age set of our patients, and enable us to propose a treatment option to avoid bone marrow transplantation.

*Data availability.* Clinical presentations are included in the supplemental material (Supplemental Table 1; supplemental material available online with this article; https://doi.org/10.1172/JCI192422DS1).

## Supplementary Material

Supplemental data

Supporting data values

## Figures and Tables

**Figure 1:**
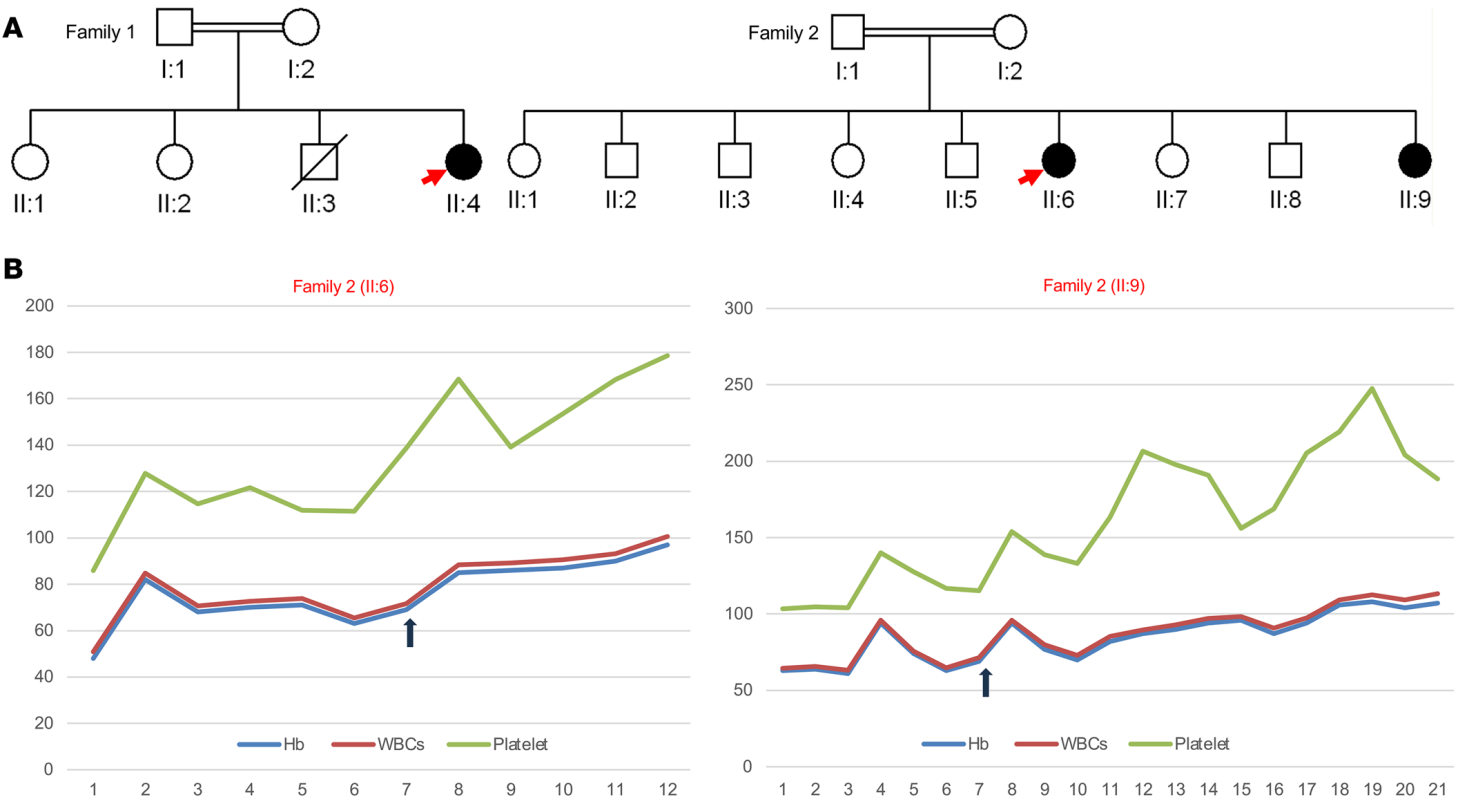
Response of OSM patients to eltrombopag. (**A**) Pedigree of the families. (**B**) Response of patients to eltrombopag (family 2; II:6 and II:7) from the seventh week.

**Table 1:**
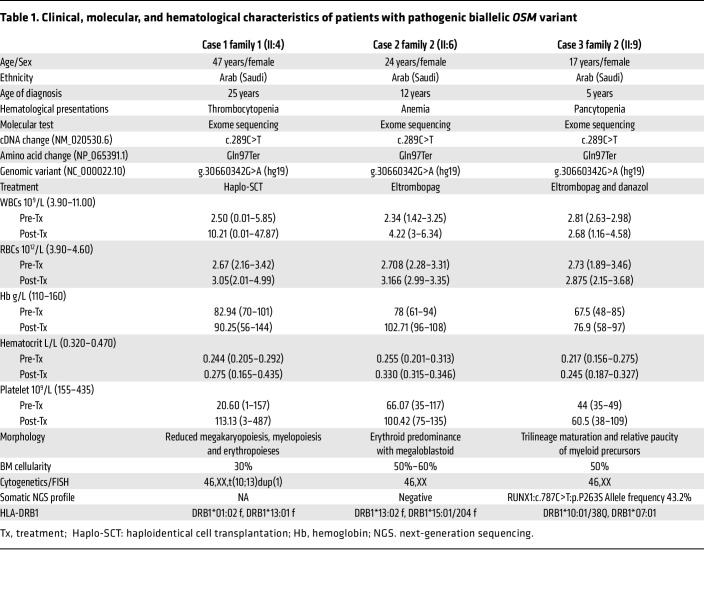
Clinical, molecular, and hematological characteristics of patients with pathogenic biallelic *OSM* variant

## Version 1 05/01/2025

Electronic publication
