# Supplementary material for: Biallelic OSM deficiency presents with juvenile myelodysplastic syndrome and response to treatment
Source: J Clin Invest. 2025 May 1;135(9):e192422. doi: 10.1172/JCI192422 (PMC12043077; doi:10.1172/JCI192422)
Supplement: Supplemental data [file jci-135-192422-s245.pdf]

Clinical phenotypes and hematological values of the three patients with a variant in *OSM*.

Family 1:

First proband (II:4) is a 46-year Saudi female with two healthy sisters and one deceased brother in his 20s due to unknown cause, her parents are second degree cousins. She was in her usual state of health till the age of 16 years when she sought medical advice due to bleeding and petechia that was attributed for her thrombocytopenia requiring platelet transfusion and later splenectomy at 21 years of age with no good response. She was later referred to a tertiary hospital due to severe aplastic anemia with short telomere in her workup that would lead to the suspicion of dyskeratosis congenita with negative molecular result for *TERC, TINF2* and *TKC* genes. She kept on Eltrombopag 150 mg daily. Despite the medical management she continued to have low platelets of 4x10^9/L requiring platelet transition once weekly. At one point steroid pulse therapy was tried with partial improvement in her platelet count that would later lead to a drop. Given the change in the karyotype with her bone marrow failure, she was thought to be progressing to Myelodysplastic syndrome (MDS) and started on folic acid, vitamin B and tranexamic acid and kept on supportive transfusion twice monthly. Admitted to the hospital electively for stem cell transplant work up where she underwent plasmapharesis followed by anti-Thymocyte globuin, intravenous immunoglobulins and Rituxmiab . Her conditioning pre-transplant included thiotepa, fludarabine, busulfan and anti-thymocyte globulin. She received her stem cell transplant from haploindentical donor with 100% donor cells Engraftment. Her Transplant was complicated with persistent refractory BK virus cystitis, CMV Viremia, GUT Graft versus host disease started, Central line sepsis, bed sore, persistent thrombocytopenia, chronic kidney disease and *Herpes Zoster* infection. On her last clinical visit 73 months post SCT she was clinically stable with a plan of regular follow up in the clinic. Her Cystitis, CMV

reaction, GUT GVHD resolved. She was noted to have persistent lymphocytosis with no clear aberrancy with a plan to exclude underlying clonality or primary vs. secondary causes.

Family 2:

Subjects 2 and 3 are siblings (II:6, II:9) who are product of consanguineous Saudi couples. The proband (II:6) is a 24 years old female. She was in her usual state of health till the age of 14 years when she started to complain of dizziness, shortness of breath, headache, bruises, bleeding and recurrent infections. She was investigated in her local hospital when she was diagnosed with anemia with multiple hospital visits for blood transfusions. However, her condition was gradually worsening as she started to show signs of pancytopenia with increased in the frequency of blood transfusions to reach once every month (from annual blood transfusions). Her bone marrow showed hypocellularity (50-60%) with reduced granulopoiesis. At that moment the differential included apalstic anemia versus myelodysplastic syndrome (MDS). She started on eltromobopag 50 mg daily and deferasirox daily. Her hemoglobin stabilized at 7.7 g/L with mild anemic symptoms. She was transfusion independent after starting the Eltromobopag. Repeated bone marrow aspiration and biopsy showed no progression, but she started to show BLAST cells on the peripheral blood smear.

Her affected sister (II:9) is 17 years old female who was in her usual state of health till 5 years of age when she started to have anemic manifestations and bleeding tendency, treated by supportive transfusion. Bone marrow biopsy was done at that time and showed no abnormalities. At age of 12 years her requirement for blood components transfusion increased, another bone marrow biopsy was done and showed hypercellular marrow with increased reticulin formation +1. Patient

kept on erythropoietin with escalating doses and reached 150 mg per2weeks, but it did not control her symptoms and became transfusion dependent. She needed at least 2 units of packed RBCs every 6 weeks. She was diagnosed with Bone marrow failure secondary to myeloproliferative disorder due to the result of bone marrow NGS showing a heterozygous variant in MPL (NM_005373.3:c.317C>T:p.Pro106Leu). Upon her referral she started on Eltrombopag 50 mg PO OD and deferasirox. She was showing response to Eltromobopag but her platelets were dropping so the dose was increased to 125 mg PO BOD with addition of Danazol 200 mg BID. Repeated Myeloid NGS showed the previously detected MPL variant, but upon review of the variant it turned out it is a common variant within the Saudi population that is associated with Thrombocythemia phenotype rather than bone marrow failure. At this stage she became transfusion independent as she required transfusion every 2-3 weeks and now 8 months follow up not requiring transfusion. However, upon the subject not-compliance to the Eltromobopag the hemoglobin started to drop again and bone marrow transplant option proposed.

Supplemental Table 1

	**Case 1 family 1 (II:4)**	**Case 2 family 2 (II:6)**	**Case 3 family 2 (II:9)**
**Age / Gender**	47 years female	24 years female	17 years female
**Age of Diagnosis**	25 years	12 years	5 years
**Hematological presentations**	Thrombocytopenia	Anemia	Pancytopenia
**Molecular test**	Whole exome sequencing	Whole exome sequencing	Whole exome sequencing
**Nucleic acid change in reference cDNA** (NM_020530.6)	c.289C>T	c.289C>T	c.289C>T
**predicted amino acid change (NP_065391.1)**	Gln97Ter	Gln97Ter	Gln97Ter
**Genomic Variant**	g.30660342G>A (hg19)	g.30660342G>A (hg19)	g.30660342G>A (hg19)

**(NC_000022.10)**			
**Treatment**	Haplo-SCT	Eltrombopag	Eltrombopag and Danazol
**WBCs 10^9/L (3.90-11.00)**			
**Pre-Tx**	2.50 (0.01-5.85)	2.34 (1.42-3.25)	2.81 (2.63-2.98)
**Post-Tx**	10.21 (0.01-47.87)	4.22 (3-6.34)	2.68 (1.16-4.58)
**RBC 10^12/L (3.90-4.60)**			
**Pre-Tx**	2.67 (2.16-3.42)	2.708 (2.28-3.31)	2.73 (1.89-3.46)
**Post-Tx**	3.05(2.01-4.99)	3.166 (2.99-3.35)	2.875 (2.15-3.68)
**Hb g/L (110-160)**			
**Pre-Tx**	82.94 (70-101)	78 (61-94)	67.5 (48-85)
**Post-Tx**	90.25(56-144)	102.71 (96-108)	76.9 (58-97)
**Hematocrit L/L (0.320-0.470)**			
**Pre-Tx**	0.244 (0.205-0.292)	0.255 (0.201-0.313)	0.217 (0.156-0.275)
**Post-Tx**	0.275 (0.165-0.435)	0.330 (0.315-0.346)	0.245 (0.187-0.327)
**MCV fL (75-95)**			
**Pre-Tx**	90.93 (84.6-102.4)	94 (87.2-106.1)	79.7 (77.8-82.5)
**Post-Tx**	89.83 (76.4-110.2)	104.4 (103.3-105.9)	86.6 (78.9-91)
**MCH pg (24.0-30.0)**			
**Pre-Tx**	30.85 (29.3-33.2)	28.71 (26.5-31.5)	24.8 (24.5-25.4)
**Post-Tx**	29.50 (25.4-35)	32.41 (31.6-33.5)	26.8 (25-28.3)
**MCHC g/L (316-360)**			
**Pre-Tx**	339.828(319-351)	305.76 (293-320)	311 (308-318)
**Post-Tx**	328.802 (299-357)	310.57 (305-318)	309.4 (294-331)
**RDW % (11-15)**			
**Pre-Tx**	23 (19.3-27.7)	24.4 (20.5-28.1)	27.2 (23.9-28.7)
**Post-Tx**	17.23 (12.7-26.5)	21 (20.1-22.8)	28.9 (22.5-35.2)
**Platelet 10^9/L (155-435)**			
**Pre-Tx**	20.60 (1-157)	66.07 (35-117)	44 (35-49)
**Post-Tx**	113.13 (3-487)	100.42 (75-135)	60.5 (38-109)
**Neutrophil Absolute Count 10^9/L (1.35-7.50)**			
**Pre-Tx**	NA	0.045 (0.02-0.09)	1.295 (1.25-1.34)
**Post-Tx**	5.11 (0.2-31.74)	0.175 (0.04-0.32)	0.99 (0.43-1.92)

**Lymphocyte Absolute (Auto) 10^9/L (1.50-4.30)**			
**Pre-Tx**	NA	5.27 (2-12)	NA
**Post-Tx**	2.4 (0.24-12.68)	6.14 (4-8)	1.38 (0.94-2.26)
**Monocyte Absolute 10^9/L (0.25-1.00)**			
**Pre-Tx**	NA	2 (1-4)	1.5 (1.37-1.62)
**Post-Tx**	14.43 (5.1-32.4)	2.858 (1-7)	1.38 (0.55-2.24)
**Eosinophil Absolute 10^9/L (0.03-1.00)**			
	NA	1.75 (1-3)	
**Pre-Tx**	0.35 (0.02-1.31)	1.17 (1-2)	NA
**Post-Tx**			0.16 (0.03-0.37)
**Basophil absolute 10^9/L (0-1.50)**			
**Pre-Tx**	NA	0.64 (0.28-0.93)	NA
**Post-Tx**	0.137 (0.01-0.45)	1.28 (0.69-1.9)	1.27 (1-2)
**MetaMyelocyte Absolute 10^9/L**			
**Pre-Tx**	NA	0.06 (0.06-0.06)	NA
**Post-Tx**	0.2 (0.01-0.71)	0.132 (0.04-0.38)	0.038 (0.02-0.06)
**Myelocyte Absolute 10^9/L**			
**Pre-Tx**	NA	0.0375 (0.03-0.06)	NA
**Post-Tx**	0.187 (0.02-1.63)	0.099 (0.05-0.19)	0.039 (0.02-0.07)
**Promyelocytes Absolute 10^9/L**			
**Pre-Tx**	NA	0.04 (0.03-0.06)	NA
**Post-Tx**	0.11 (0.02-0.4)	0.12 (0.04-0.32)	1.28 (1-3)
**BLAST Absolute**			
**Pre-Tx**	NA	NA	NA
**Post-Tx**	NA	0.13 (0.07-0.23)	NA

**morphology**	reduced megakaryopoiesis, myelopoiesis and erythropoieses	erythroid predominance with megaloblastoid	trilineage maturation and relative paucity of myeloid precursors
**BM cellularity**	30%	50-60%.	50%
**Cytogenetics/FISH**	46,XX,t(10;13)dup(1)	46,XX	46,XX
**Somatic NGS profile**	NA	Negative	*RUNX1*:c.787C>T:p.P263S Allele frequency 43.2%
**HLA-DRB1**	DRB1*01:02 f, DRB1*13:01 f	DRB1*13:02 f, DRB1*15:01/204 f	DRB1*10:01/38Q, DRB1*07:01

Acknowledgements:

The authors thank the patients, their family for their participation in this study and core facility in GMCoE, King Faisal Specialist Hospital and Research Center, Riyadh. The study approved under IRB-approved research protocol (KFSHRC RAC #2241173).
